# Supplementary material for: The Effectiveness of Dog Population Management: A Systematic Review
Source: Animals (Basel). 2019 Nov 22;9(12):1020. doi: 10.3390/ani9121020 (PMC6940938; doi:10.3390/ani9121020)
Supplement: Supplementary file 1 [file animals-09-01020-s001.pdf]

Supplementary information

Table S1. Key words and combinations. The columns represent the key word categories (separated by "AND" in the search) and rows display word variations (separated by "OR" in the search).

Key word	1	2	3	4
	AND		AND	AND
	"Dog”	Stray*	Kill*	“Population”
	“Dogs"	“Feral"	Cull*	Control*
	“Canine”	Untame*	Euthan*	Restrain*
	“Canines”	Undomesticat*	Destroy*	Constrain*
		“Street"	Extermin*	Limit*
		Free-roam* (free-roaming)	Execut*	Restrict*
		Roam* (roaming)	Slaughter*	Manag*
		Unrestrict*	Terminat*	Dynamic*
		Free-rang*	“Lethal"	“Ecology”
		Rang*	Shelter*	Demograph*
		Abandon*	Rehom*	
		Unrestrain*	“Sanctuary”	
		Unconfin*	Adopt*	
			Rescue centre	
			Neuter*	
			Sterili* (sterilise)	
			Infertil*	
			Fert* (fertility)	
			Reproduc*	
			Breed*	
			Desex*	
			Castrat*	
			Contracept*	
			Birth*	
			Spay	

Inter-observer reliability:

The inter-observer reliability check resulted in 97% (146/150) agreement in stage one; and 60% (18/30) agreement at stage two.

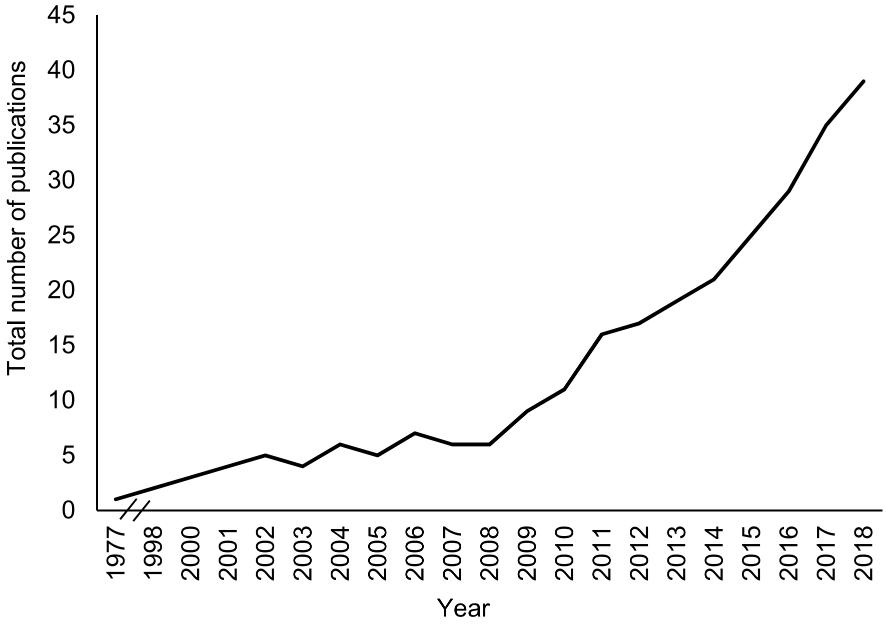

Figure S1. Cumulative number of publications in the final corpus per year between 1977 and 2018. Note the break in year from 1977 to 1998.

Table S2. Papers in the final corpus by subject, dog population management method, country, economic status of that country, and funding organisation.

Author	Dog population management method	Country	Continent	Geographical Region	Economy status*	Funding type/driving organisation**
[1]	Neutering (CNR)	India	Asia	Asia	Lower middle income	Charity and University
[2]	Neutering (CNR) and Culling (indiscriminate)	USA	North America	North America	High income	Government and University
[3]	Neutering (CNR) and neutering (owned) and sheltering (rehoming)	Multiple	Multiple	Multiple	Multiple	Government and University
[4]	Neutering (owned)	Brazil	South America	South America	Upper middle income	Charity, Government, and University
[5]	Neutering (CNR) and sheltering	Italy	Europe	European Union	High income	Not reported
[6]	Neutering (CNR) and sheltering	Brazil	South America	South America	Upper middle income	Government and University
[7]	Culling (indiscriminate)	Multiple	No specific	No specific	No specific	University
[8]	Neutering (CNR) and sheltering	Canada	North America	North America	High income	Government and University
[9]	Immunocontraceptive & culling (indiscriminate)	Multiple	No specific	No specific	No specific	Government and University

[10]	Neutering (CNR)	Brazil	South America	South America	Upper middle income	University
[11]	Sheltering (rehoming)	Turkey	Europe/Asia	Europe	Upper middle income	University
[12]	Neutering (undefined) and Culling (indiscriminate)	Cyprus	Europe	European Union	High income	Government
[13]	Neutering (CNR)	India	Asia	Asia	Lower middle income	Charity, Government and University
[14]	Neutering (CNR) and neutering (owned) and sheltering (rehoming)	USA	North America	North America	High income	Charity
[15]	Neutering (CNR)	Brazil	South America	South America	Upper middle income	Charity, Government and University
[16]	Neutering (CNR)	India	Asia	Asia	Lower middle income	Charity
[17]	Neutering (CNR) and sheltering	Italy	Europe	European Union	High income	Government and University
[18]	Culling (indiscriminate)	China	Asia	Asia	Upper middle income	Government and University
[19]	Sheltering	Spain	Europe	European Union	High income	Government
[20]	Neutering (owned) and owned dog confinement	Mexico	South America	Central America	Upper middle income	University
[21]	Neutering (CNR) and Culling (indiscriminate)	Sri Lanka	Asia	Asia	Upper middle income	Government

[22]	Culling (indiscriminate)	No specific	No specific	No specific	No specific	University
[23]	Taxation	No specific	No specific	No specific	No specific	University
[24]	Culling (infected dogs)	Brazil	South America	South America	Upper middle income	Government and University
[25]	Neutering (undefined) & Sheltering	Thailand	Asia	Asia	Upper middle income	Government and University
[26]	Neutering (owned) and Culling (indiscriminate)	Cyprus	Europe	European Union	High income	Government
[27]	Neutering (CNR) & Sheltering (transportation of dogs)	Serbia	Europe	Europe	Economy in transition	University
[28]	Neutering (CNR)	India	Asia	Asia	Lower middle income	Charity
[29]	Neutering (CNR)	India	Asia	Asia	Lower middle income	Government and Charity
[30]	Neutering (owned) and sheltering	Canada	North America	North America	High income	Charity and University
[31]	Neutering (CNR)	Bangladesh	Asia	Asia	Lower middle income	Charity and Government
[32]	Neutering (CNR)	India	Asia	Asia	Lower middle income	Charity and University
[33]	Neutering (CNR)	India	Asia	Asia	Lower middle income	Charity and University
[34]	Neutering (CNR)	India	Asia	Asia	Lower middle income	Charity and University
[35]	Neutering (CNR) and Culling (indiscriminate)	India	Asia	Asia	Lower middle income	Charity and University

[36]	Neutering and waste management	India	Asia	Asia	Lower middle income	University
[37]	Culling (infected dogs)	Brazil	South America	South America	Upper middle income	Charity and University
[38]	Culling (indiscriminate)	China	Asia	Asia	Upper middle income	Government and University
[39]	Culling (indiscriminate)	Chad	Africa	Africa	Low income	Charity and Government

* Economy status defined by The World Bank 2019 country income classification [40].

** Includes funding organisation and author affiliations.

animals

MDPI

Table S3. Number (and ratio) of published articles from the final corpus that measure the impact of the management method(s) studied.

Impact Measure	No. Papers	
**Dog Health & Welfare**		
Fertility control	4	(10.3%)
Fertility control and sheltering	1	(2.6%)
Sheltering	1	(2.6%)
**Dog Demographics**		
Fertility control	4	(10.3%)
Fertility control and culling	2	(5.1%)
Fertility control and movement restriction	1	(2.6%)
Fertility control and sheltering	5	(12.8%)
Taxation	1	(2.6%)
**Public Attitude**		
Fertility control	1	(2.6%)
Fertility control and sheltering	2	(5.1%)
**Public Health**		
Culling	7	(17.9%)
Fertility control	3	(7.7%)
Fertility control and culling	4	(10.3%)
Fertility control and sheltering	1	(2.6%)
Sheltering	1	(2.6%)
**Wildlife**		
Fertility control	1	(2.6%)
**Total Papers**	39	(100%)

Table S4. Number of papers investigating combinations of dog populations.

Dog population under investigation	Number of papers from final corpus investigating dog population
Only free-roaming owned dogs	1 (2.6%)
Only free-roaming unowned dogs	5 (12.8%)
Free-roaming unowned and free-roaming owned	19 (48.7%)
Free-roaming unowned, free-roaming owned and restricted owned	7 (17.9%)
Free-roaming unowned, free-roaming owned, restricted owned and shelter dogs	3 (7.7%)
Free-roaming unowned and shelter dogs	1 (2.6%)
Free-roaming owned and restricted owned	1 (2.6%)
Undefined	2 (5.1%)

Table S5. Summary of the reporting of study quality indicators in final corpus (excluding modelling studies). X indicates no metric was reported, ✓ indicates metric was reported and – indicates metric was not applicable for the study type.

Paper	Reporting of power calculation	Reporting of sample size calculation	Control population included	Reporting of inter-observer reliability	Reporting of baseline characteristics	Reporting quality indicator score
[19]	X	X	X	-	X	0% (0/4)
[32]	✓	✓	✓	-	-	100% (3/3)
[28]	X	X	X	-	X	0% (0/4)
[33]	X	X	X	X	✓	20% (1/5)
[34]	X	X	✓	✓	-	25% (1/4)
[31]	-	-	X	-	-	0% (0/1)
[21]	X	X	X	-	✓	25% (1/4)
[6]	-	-	✓	X	✓	67% (2/3)
[1]	X	X	X	✓	✓	40% (2/5)
[16]	X	X	X	-	-	0% (0/3)
[29]	X	X	X	✓	✓	40% (2/5)
[10]	✓	✓	X	✓	✓	80% (4/5)
[11]	X	X	X	-	-	0% (0/3)
[12]	X	X	X	-	✓	20% (1/4)
[26]	X	X	X	-	✓	20% (1/4)
[5]	X	X	X	-	-	0% (0/3)
[30]	X	X	X	-	✓	20% (1/4)
[25]	-	-	X	-	X	0% (0/2)
[8]	X	X	X	✓	-	20% (1/4)
[27]	X	X	X	-	-	0% (0/3)
[15]	-	-	X	-	✓	50% (1/2)
[24]	X	X	X	-	✓	20% (1/4)

[37]	X	X	✓	-	✓	50% (2/4)
Percentage reporting study quality indicators [a]	11%	11%	17%	71%	80%	

[a] Calculated as a percentage of those studies where this quality indicator is applicable.

References

1. Airikkala-Otter, I.; Gamble, L.; Mazeri, S.; Handel, I.G.; Bronsvoort, B.M. de C.; Mellanby, R.J.; Meunier, N. V Investigation of short-term surgical complications in a low-resource, high-volume dog sterilisation clinic in India. *BMC Vet. Res.* **2018**, *14*, 1–8.
2. Amaku, M.; Dias, R.A.; Ferreira, F. Dynamics and Control of Stray Dog Populations. *Math. Popul. Stud.* **2010**, *17*, 69–78.
3. Santos Baquero, O.; Akamine, L.A.; Amaku, M.; Ferreira, F. Defining priorities for dog population management through mathematical modeling. *Prev. Vet. Med.* **2016**, *123*, 121–127.
4. Santos Baquero, O.; Marconcin, S.; Rocha, A.; Maria Garcia, R. de C. Companion animal demography and population management in Pinhais, Brazil. *Prev. Vet. Med.* **2018**, *158*, 169–177.
5. Barnard, S.; Chincarini, M.; Di Tommaso, L.; Di Giulio, F.; Messori, S.; Ferri, N. Free-roaming dogs control activities in one italian province (2000-2013): is the implemented approach effective? *Maced. Vet. Rev.* **2015**, *38*, 149–158.
6. Belo, V.S.; Struchiner, C.J.; Werneck, G.L.; Teixeira Neto, R.G.; Tonelli, G.B.; de Carvalho Junior, C.G.; Nascimento Ribeiro, R.A.; da Silva, E.S. Abundance, survival, recruitment and effectiveness of sterilization of free-roaming dogs: A capture and recapture study in Brazil. *PLoS One* **2017**, *12*, 1–19.
7. Bhunu, C.P. Impact of culling stray dogs and vaccination on the control of human rabies: a mathematical modeling approach. *Int. J. Biomath.* **2011**, *4*, 379–397.
8. Boey, J. Working with communities to improve the quality of life of British Columbia's free-roaming dogs and their people. Thesis., University of Victoria, 2017.
9. Carroll, M.J.; Singer, A.; Smith, G.C.; Cowan, D.P.; Massei, G. The use of immunocontraception to improve rabies eradication in urban dog populations. *Wildl. Res.* **2010**, *37*, 676–687.
10. Costa, E.D.; Martins, C.M.; Cunha, G.R.; Catapan, D.C.; Ferreira, F.; Oliveira, S.T.; Maria Garcia, R. de C.; Biondo, A.W. Impact of a 3-year pet management program on pet population and owner's perception. *Prev. Vet. Med.* **2017**, *139*, 33–41.
11. Demirbas, Y.S.; Emre, B.; Kockaya, M. Integration ability of urban free-ranging dogs into adoptive families' environment. *J. Vet. Behav. Appl. Res.* **2014**, *9*, 222–227.
12. Economides, P.; Christofi, G. Evaluation of control programmes for echinococcosis/hydatidosis in Cyprus. *Rev. Sci. Tech. L Off. Int. DES Epizoot.* **2000**, *19*, 784–792.
13. Fitzpatrick, M.C.; Shah, H.A.; Pandey, A.; Bilinski, A.M.; Kakkar, M.; Clark, A.D.; Townsend, J.P.; Abbas, S.S.; Galvani, A.P. One Health approach to cost-effective rabies control in India. *Proc. Natl. Acad. Sci. U. S. A.* **2016**, *113*, 14574–14581.
14. Frank, J. An interactive model of human and companion animal dynamics: the ecology and

economics of dog overpopulation and the human costs of addressing the problem. *Hum. Ecol.* **2004**, *32*, 107–130.

15. Garcia, R.C.M.; Amaku, M.; Biondo, A.W.; Ferreira, F. Dog and cat population dynamics in an urban area: evaluation of a birth control strategy. *Pesqui. Vet. Bras.* **2018**, *38*, 511–518.
16. Hiby, L.R.; Reece, J.F.; Wright, R.; Jaisinghani, R.; Singh, B.; Hiby, E.F. A mark-resight survey method to estimate the roaming dog population in three cities in Rajasthan, India. *BMC Vet. Res.* **2011**, *7*, 1–9.
17. Hogasen, H.R.; Er, C.; Di Nardo, A.; Dalla Villa, P.; Høgåsen, H.; Er, C.; Nardo, A. Di; Villa, P.D. Free-roaming dog populations: A cost-benefit model for different management options, applied to Abruzzo, Italy. *Prev. Vet. Med.* **2013**, *112*, 401–413.
18. Hou, Q.; Jin, Z.; Ruan, S. Dynamics of rabies epidemics and the impact of control efforts in Guangdong Province, China. *J. Theor. Biol.* **2012**, *300*, 39–47.
19. Jimenez, S.; Perez, A.; Gil, H.; Schantz, P.M.; Ramalle, E.; Juste, R.A. Progress in control of cystic echinococcosis in La Rioja, Spain: decline in infection prevalences in human and animal hosts and economic costs and benefits. *ACTA Trop.* **2002**, *83*, 213–221.
20. Kisiel, L. Using a dog demography field study to inform the development of an agentbased computer simulation. Evaluating owned dog population control interventions in a small, semi-urban community in Mexico. Thesis., The University of Guelph, 2017.
21. Kumarapeli, V.; Awerbuch-Friedlander, T. Human rabies focusing on dog ecology-A challenge to public health in Sri Lanka. *ACTA Trop.* **2009**, *112*, 33–37.
22. Leung, T.; Davis, S.A. Rabies Vaccination Targets for Stray Dog Populations. *Front. Vet. Sci.* **2017**, *4*, 1–10.
23. Liu, S.; Chen, H. Solving Stray-Animal Problems by Economic Policies. *Taipei Econ. Inq.* **2016**, 1–27.
24. Melo, S.N.; Teixeira-Neto, R.G.; Werneck, G.L.; Struchiner, C.J.; Ribeiro, R.A.N.; Sousa, L.R.; de Melo, M.O.G.; Carvalho Junior, C.G.; Penaforte, K.M.; Manhani, M.N.; et al. Prevalence of visceral leishmaniasis in A population of free-roaming dogs as determined by multiple sampling efforts: A longitudinal study analyzing the effectiveness of euthanasia. *Prev. Vet. Med.* **2018**, *161*, 19–24.
25. Panichabhongse, P. The epidemiology of rabies in Thailand. Thesis, Massey University, 2001.
26. Polydorou, K. The anti-echinococcosis campaign in Cyprus. *Trop. Anim. Health Prod.* **1977**, *9*, 141–146.
27. Radisavljevic, K.; Vucinic, M.; Becskei, Z.; Stanojkovic, A.; Ostovic, M. Comparison of stress level indicators in blood of free-roaming dogs after transportation and housing in the new environment. *J. Appl. Anim. Res.* **2017**, *45*, 52–55.
28. Reece, J.F.; Chawla, S.K. Control of rabies in Jaipur, India, by the sterilisation and vaccination of neighbourhood dogs. *Vet. Rec.* **2006**, *159*, 379–383.

29. Reece, J.F.; Chawla, S.K.; Hiby, A.R. Decline in human dog-bite cases during a street dog sterilisation programme in Jaipur, India. *Vet. Rec.* **2013**, *172*, 473–477.
30. Schurer, J.M.; Phipps, K.; Okemow, C.; Beatch, H.; Jenkins, E. Stabilizing Dog Populations and Improving Animal and Public Health Through a Participatory Approach in Indigenous Communities. *Zoonoses Public Health* **2015**, *62*, 445–455.
31. Tenzin, T.; Ahmed, R.; Debnath, N.C.; Ahmed, G.; Yamage, M. Free-Roaming Dog Population Estimation and Status of the Dog Population Management and Rabies Control Program in Dhaka City, Bangladesh. *PLoS Negl. Trop. Dis.* **2015**, *9*, 1–14.
32. Totton, S.C.; Wandeler, A.I.; Ribble, C.S.; Rosatte, R.C.; McEwen, S.A. Stray dog population health in Jodhpur, India in the wake of an animal birth control (ABC) program. *Prev. Vet. Med.* **2011**, *98*, 215–220.
33. Totton, S.C.; Wandeler, A.I.; Zinsstag, J.; Bauch, C.T.; Ribble, C.S.; Rosatte, R.C.; McEwen, S.A. Stray dog population demographics in Jodhpur, India following a population control/rabies vaccination program. *Prev. Vet. Med.* **2010**, *97*, 51–57.
34. Yoak, A.J.; Reece, J.F.; Gehrt, S.D.; Hamilton, I.M. Disease control through fertility control: Secondary benefits of animal birth control in Indian street dogs. *Prev. Vet. Med.* **2014**, *113*, 152–156.
35. Yoak, A.J.; Reece, J.F.; Gehrt, S.D.; Hamilton, I.M. Optimizing free-roaming dog control programs using agent-based models. *Ecol. Modell.* **2016**, *341*, 53–61.
36. Belsare, A. V.; Gompper, M.E. A model-based approach for investigation and mitigation of disease spillover risks to wildlife: Dogs, foxes and canine distemper in central India. *Ecol. Modell.* **2015**, *296*, 102–112.
37. Ashford, D.; David, J.; Freire, M.; David, R.; Sherlock, I.; Da Conceicao, M.; Sampaio, D.; Badaro, R. Studies on control of visceral leishmaniasis: Impact of dog control on canine and human visceral leishmaniasis in Jacobina, Bahia, Brazil. *Am. J. Trop. Med. Hyg.* **1998**, *59*, 53–57.
38. Zhang, J.; Jin, Z.; Sun, G.Q.; Zhou, T.; Ruan, S. Analysis of rabies in China: transmission dynamics and control. *PLoS One* **2011**, *6*, 1–9.
39. Zinsstag, J.; Durr, S.; Penny, M.; Mindekem, R.; Roth, F.; Gonzalez, S.; Naissengar, S.; Hattendorf, J. Transmission dynamics and economics of rabies control in dogs and humans in an African city. *Proc. Natl. Acad. Sci.* **2009**, *106*, 14996–15001.
40. The World Bank - Countries and Economies Available online: https://data.worldbank.org/country.
